# Direct Observation of Raman Spectra in Black Phosphorus under Uniaxial Strain Conditions

**DOI:** 10.3390/nano9040566

**Published:** 2019-04-08

**Authors:** Stacy Liang, Md Nazmul Hasan, Jung-Hun Seo

**Affiliations:** Department of Materials Design and Innovation, University of Buffalo, Buffalo, NY 14260, USA; stacyli@buffalo.edu (S.L.); mhasan9@buffalo.edu (M.N.H.)

**Keywords:** black phosphorus, uniaxial strain, flexible substrate

## Abstract

In this paper, we systematically studied the Raman vibration of black phosphorus (BP) transferred onto a germanium (Ge)-coated polydimethylsiloxane (PDMS) substrate, which generates a much higher contrast in BP. This engineered flexible substrate allowed us to directly observe a much thinner BP layer on the flexible substrate at the desired location. Therefore, it enabled us to perform Raman spectroscopy immediately after exfoliation. The Raman spectra obtained from several BP layers with different thicknesses revealed that the clear peak shifting rates for the A_g_^1^, B_2g_, and A_g_^2^ modes were 0.15, 0.11, and 0.11 cm^−1^/nm, respectively. Using this value to identify a 2–3-layered BP, a study on the strain–Raman spectrum relationship was conducted, with a maximum uniaxial strain of 0.89%. The peak shifting of A_g_^1^, B_2g_, and A_g_^2^ caused by this uniaxial strain were measured to be 0.86, 0.63, and 0.21 cm^−1^/Δε, respectively.

## 1. Introduction

Strain engineering has been known as an effective way to modulate the electronic, transport, and optical properties of semiconductors [1,2,3,4]. This method is particularly powerful when engineering low-dimensional semiconductors, such as one- and two-dimensional semiconductors (1D and 2D, respectively), since these low-dimensional semiconductors can tolerate much higher strain levels than three-dimensional (3D) semiconductors, such as bulk or thin-films [5,6]. For example, graphene is known to sustain strains of up to 15% without any noticeable damage to its crystalline structure [6,7]. As a result, strain engineering is a viable way to tune low-dimensional semiconductors’ electrical, optical, chemical, and mechanical performances [8,9,10,11,12].

Recently, black phosphorus (BP, also known as phosphorene) was mechanically exfoliated from its bulk format [13,14,15]. Unlike the widely studied graphene, BP exhibits a finite and direct band gap varying from 0.5 eV in bulk to ~1.2 eV for a single layer, and its free-carrier mobility (approximately 1000 cm^2^/v·s) is better than that of other typical 2D semiconductors, such as molybdenum sulfate (MoS_2_; approximately 200 cm^2^/v·s) [16,17,18,19]. As a result, various optoelectronic applications such as photodetectors and field-effect transistors, have been recently demonstrated [20,21,22,23]. Another attractive property of BP is its wide range of band gap tunability by mechanical strain. It is predicted that the band gap of BP can be modulated from as low as 0.55 eV up to 1.1 eV, while still maintaining a direct band gap by the application of ±8% of mechanical strain [24,25,26]. Such a wide tunability in the band gap of BP from 0.55 eV to 1.1 eV corresponds to the 1000 nm to 2200 nm wavelength range, suggesting that BP can be used as an active material for the near-infrared tunable light source. For this reason, there have been several theoretical and empirical attempts to demonstrate the characteristics of BP under strain conditions [27,28]. However, most of the experiments on the strain properties of BP have used thick BP (>10 nm thickness), because the visibility of BP decreases dramatically as it becomes thinner (similar to other 2D materials). For example, 13 nm- and 15 nm-thick BP (>20 layers) were used in the studies by Zhu et al. to examine the mechanical robustness of flexible BP devices under bending conditions. Material characterization studies of few-layered BP directly on a flexible substrate under strain conditions have not been performed so far.

In this paper, we investigated the strain dependence of the Raman characteristics of BP, taken directly from a flexible polymer substrate, enabled by a thin layer of germanium (Ge) coated on the backside of the polymer. This thin Ge contrast booster reflector on the backside of the polymer substrate provided a much higher contrast in BP and allowed us to investigate BP at the desired location with specific thicknesses. Also, our structure allowed us to perform Raman spectroscopy immediately after exfoliation and to perform Raman characterization faster avoiding the structural degradation caused by environmental factors, such as oxidation, which can potentially cause unintended Raman shifts.

## 2. Materials and Methods

**Figure 1:**
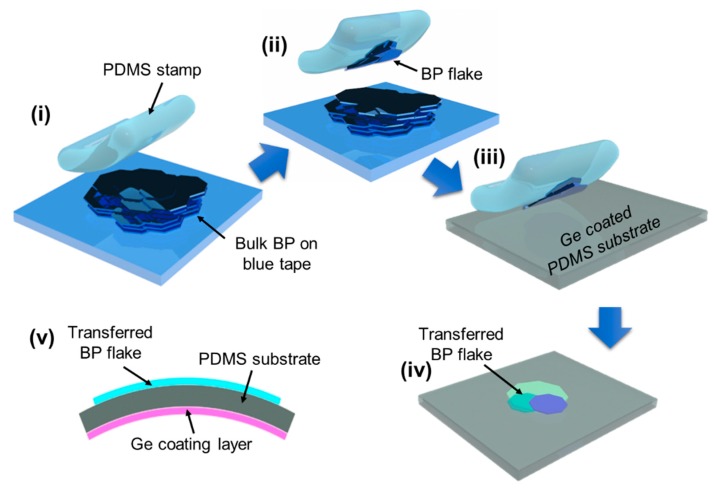
A schematic illustration of the sample preparation process on a Ge-coated polydimethylsiloxane (PDMS) substrate. (**i**) Mechanical exfoliation of black phosphorus (BP) flakes from bulk BP using blue tape, (**ii**) pick-up process of exfoliated BP flakes from the blue tape, (**iii**,**iv**) a transfer process of BP flakes onto the Ge-coated PDMS substrate, (**v**) cross-sectional view of the final structure under bending conditions.

Figure 1 shows a schematic illustration of the preparation of the BP sample. The BP sample (purity of 99.9999%) was purchased from 2D Semiconductors USA Inc (Scottsdale, AZ, USA). The process started with mechanical exfoliation from the bulk BP using a well-known micromechanical cleavage technique (also known as the “Scotch-tape” method), which is commonly used to create other 2D semiconductors from their bulk formats (Figure 1(i)) [29]. In this step, thin layers of BP that were a few nanometers thick were obtained. The thin BP layers were then carefully placed onto an ultrathin (>30 um) polydimethylsiloxane (PDMS) substrate prepared by spin-coating on a Petri dish (Figure 1(ii,iii)). Prior to the BP transfer step, a thin Ge layer (200nm) was deposited on the backside of the PDMS substrate using an e-beam evaporation method, which significantly enhanced the reflection of BP on the PDMS substrate. As shown in Figure 1(iv), once the transfer process was completed, the samples were immediately characterized under different strain conditions within 10 min to avoid unwanted BP degradation. Also, we prepared new samples each time we performed Raman spectroscopy under different strain conditions to maintain a high BP quality.

## 3. Results and Discussion

### 3.1. Structure Analysis by Optical Simulation

**Figure 2:**
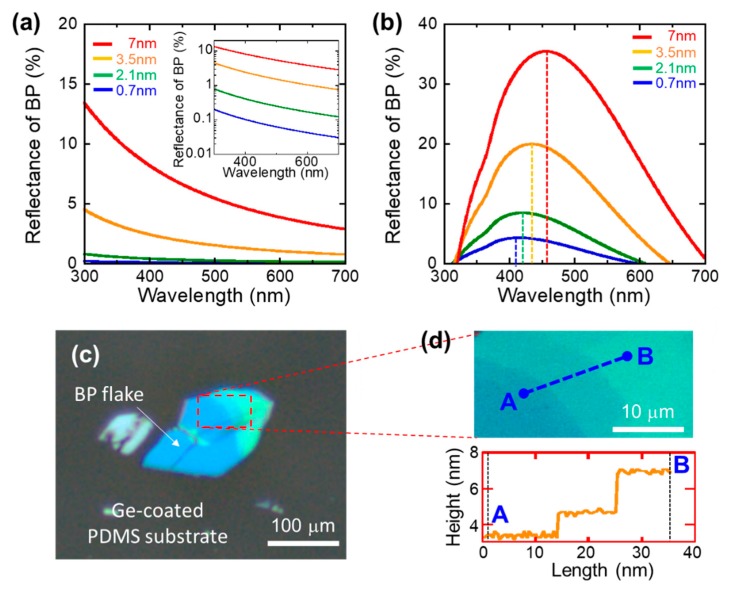
Simulated reflectance of BP on (**a**) a typical PDMS substrate (~30 um) and (**b**) a Ge-coated PDMS substrate (30 um + 200 nm). The inset of Figure 2a is the log-scale reflectance of BP to show the details of reflectance. (**c**) Microscopic image showing typical BP flakes on a Ge-coated PDMS substrate. (**d**) (lower) AFM surface profile between point A and B. (upper) The scanned area is shown.

In most of the 2D materials, it is well known that mono- or few-layered 2D materials are visible only when they are transferred onto a specific substrate that has particular refractive index and dielectric constant. Figure 2a,b show the relationship between the simulated reflectance versus wavelength of BP on a thin PDMS substrate (with and without Ge-coating on the PDMS substrate) as a function of BP thickness. To simulate the contrast enhancement of the BP layer obtained by employing the Ge-coated PDMS substrate and compare it with that of the reference bare PDMS substrate, we calculated the reflection of the multi-layered structure (BP/PDMS/Ge) using the Fresnel equation and Snell’s equation [30]. The light reflected by an interface is determined by the discontinuity components of the two materials. For multiple interfaces, the total amount of reflected light is the sum of individual reflections. Depending upon their phase relationships, the reflections from the interfaces can be calculated using Equation (1) [30]:

(1) $$R=\frac{{\left(n-2\right)}^{2}+k^{2}}{{\left(n+2\right)}^{2}+k^{2}}$$

where *n* is the refractive index, and *k* is the absorptance of the film. The optical contrast (*C_λ_*) is the fractional change of reflection because of the presence of the Ge layer on the substrate and is defined by Equation (2) [31]:

(2) $$C_{\lambda }=\frac{R_{BPonPDMS}-R_{Ge+BPonPDMS}}{R_{Ge+BPonPDMS}}$$

where *R_Ge + BPonPDMS_* and *R_BPonPDMS_* are the reflected intensities collected from the Ge-coated PDMS substrate and the reference PDMS substrate, respectively. Therefore, when we designed the substrate structure, we carefully checked the reflective index of each layer, since the visibility (i.e., the contrast of BP) can be enhanced or reduced depending on the reflective index (*n*). In our case, the refractive indices of 3.1 and 1.4 for BP and PDMS, respectively, were used in the calculation. However, any materials that have a high-reflective index, such as Si (*n_Si_* = 4.1), can also enhance the contrast of BP, although Ge has better optical and process advantages (higher reflective index and easier to deposit at a lower temperature) compared to Si. The simulated reflectance clearly indicated that the reflectance of BP was nearly invisible (<5%) when the thickness of the BP was less than 3.5 nm (i.e., five layers), which agrees well with experimental observations [19]. On the other hand, as shown in Figure 2b, the simulated reflectance of BP on a thin Ge-coated PDMS substrate showed a significantly enhanced reflectance, namely, 37%, 20%, 9%, and 4.5% for 7, 3.5, 2.1, and 0.7 nm-thick BP, respectively. Interestingly, the peak wavelength shifted to a shorter wavelength as the thickness of BP was reduced. For example, the peak wavelength that appeared at 480 nm for the 7 nm-thick BP appeared at 410 nm for the 0.7 nm-thick BP. This simulated color shift was also observed in our experiment, as shown in Figure 2c. As shown in Figure 2d, an atomic force microscopy (AFM) analysis was carried out using a Bruker AFM system with non-contact mode from the 20 mm × 20 mm area to prevent any possible damage to the sample during surface profiling. The layer thickness profile also matched well with the simulated thickness–color relationship. In other words, the wavelength changed depending on the refractive index and thickness of the thin film: 2·*t* = *m*·λ/*n*_film_, where λ is the wavelength of the reflected light, and m is an integer. When the refractive index is fixed, as in our case, this equation can be rewritten as 2·*t* = *m*·λ/*n*_film_, which shows a proportional relationship between the thickness of a thin film (*t*) and the wavelength (λ). As simulated and experimentally verified, we observed the wavelength shift to a lower wavelength as the thickness of the BP layer decreased. This thickness–wavelength shifting relationship was stronger when the refractive index was higher.

Therefore, it is clear that the thin Ge-coated PDMS substrate improved the reflectance of BP enough to directly observe its crystal shapes under the microscope. Such high contrast also allowed us to find the same location in Raman spectroscopy during strain characterization, as described below.

### 3.2. Characterization of the Relationship between Raman Spectraand Thickness in BP

**Figure 3:**
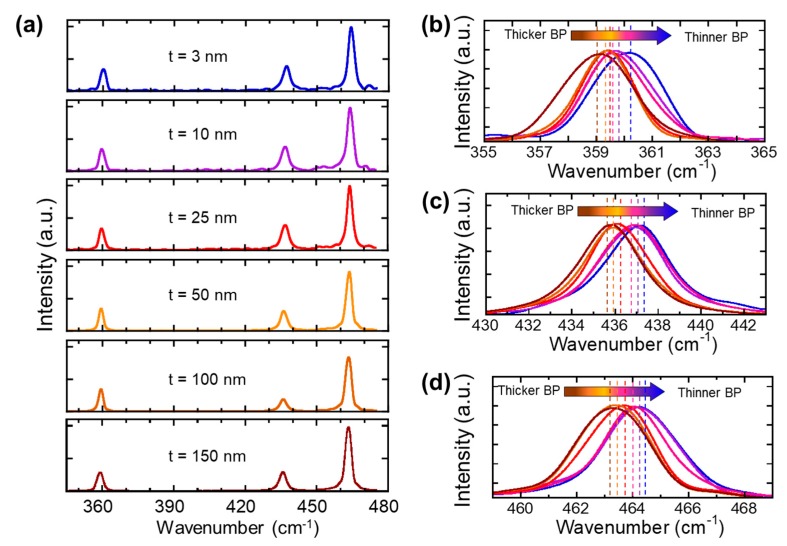
(**a**) Raman spectra of BP taken at different thicknesses from 150 nm to 3 nm. (**b**–**d**) Magnified Raman spectra of the A_g_^1^, B_2g_, and A_g_^2^ Raman modes as a function of their thickness.

**Figure 4:**
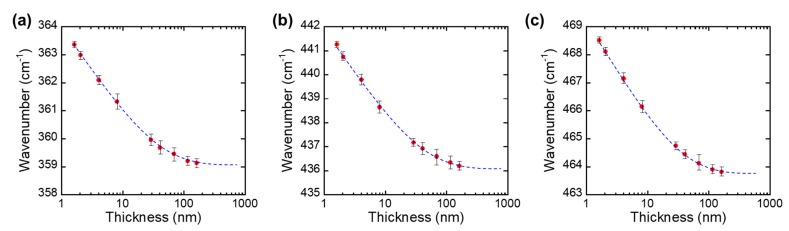
Peaks of (**a**) A_g_^1^, (**b**) B_2g_, and (**c**) A^2^_g_ Raman modes as a function of their thickness.

In order to investigate the crystalline quality of BP, Raman spectroscopy was performed using Renishaw Raman spectroscopy. The excitation was provided by a linearly polarized 514 nm excitation laser along a zigzag direction with a 50× objective lens. The diameter of the laser spot was 1 µm. In order to avoid BP ablation caused by laser-induced heating, all Raman spectra were recorded at a low laser power (200 uW) with an exposure time of 10 s and accumulations of 10 times. Figure 3a shows the Raman spectra of transfer-printed BP ranging from 150 nm to 3 nm on a Ge-coated PDMS substrate. Figure 3a represents the Raman spectra of BP layers with different thicknesses ranging from 360 cm^−1^ to 480 cm^−1^. In each spectrum, three Raman modes were present at 360 cm^−1^, 436 cm^−1^, and 464 cm^−1^, each of which was assigned a unique phonon mode of BP: (1) A_g_^1^, (2) B_2g_, (3) and A_g_^2^ [19,32,33]. Since the observed BP phonon modes matched the phonon modes seen in their bulk form (centered at 361 cm^−1^, 438 cm^−1^, and 466 cm^−1^), the Raman spectra confirmed that the lattice of BP was retained during the exfoliation step. Also, when we performed Raman spectroscopy, we investigated the edge of the BP flake and confirmed the armchair direction by comparing the relative peak intensity of the A_g_^1^, B_2g_, and A_g_^2^ phonon modes. Once we confirmed the direction of the BP edge, the sample was rotated 90 degrees and attached to the metal mold to perform a Raman spectroscopy under bending conditions. Figure 3b–d shows each phonon mode of BP as its thickness was reduced from 150 nm to 3 nm. All of the Raman phonon modes (A_g_^1^, B_2g_, and A_g_^2^) demonstrated blue-shifting as the thickness increased. Figure 3b–d presents the overlay peak positions of the A_g_^1^, B_2g_, and A_g_^2^ phonon modes as a function of the wavenumber, which showed a noticeable peak shifting. Figure 4a–c represents the trend in A_g_^1^, B_2g_, and A_g_^2^ Raman peaks as a function of their thickness. The blue dotted lines in Figure 4 show the polynomial extrapolation of the measured data points. The peak wavenumbers of the A_g_^1^, B_2g_, and A_g_^2^ modes reached 364 cm^−1^, 442 cm^−1^, and 469 cm^−1^, as BP became a monolayer. The peak positions of the A_g_^1^, B_2g_, and A_g_^2^ modes gradually decreased until the thickness of BP reached about 40–50 nm. As shown in Figure 4, all the A_g_^1^, B_2g_, and A_g_^2^ phonon modes were saturated when thicker than 40–50 nm; therefore, the BP thickness of 40–50 nm was a transition point at which the bulk property became dominant. We also noticed that the three Raman modes (A_g_^1^, B_2g_, and A_g_^2^) had different sensitivities to their thicknesses; in other words, the peak shifting rates for the A_g_^1^, B_2g_, and A_g_^2^ modes were 0.15 cm^−1^/nm, 0.11cm^−1^/nm, and 0.11cm^−1^/nm, respectively. The slightly higher shifting rate in the A_g_^1^ mode could be explained by the stiffer Ag^1^ vibration with increasing BP thickness. Therefore, the peak distance between the A_g_^1^ modes and B_2g_ or A_g_^2^ modes, namely, the difference in their frequencies (Δω), could be used as an effective thickness indicator in order to examine the thickness of BP layers.

### 3.3. Characterization of the Raman vs Strain Relationship of BP

**Figure 5:**
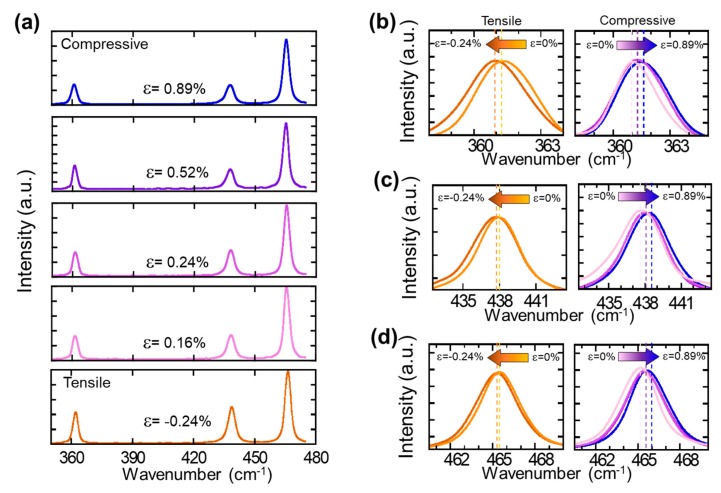
(**a**) Raman spectra of BP measured under uniaxial strains of up to 0.89% of the tensile strain (for the convex mold) and up to 0.24% of the compressive strain. (**b**–**d**) Magnified Raman spectra of the A_g_^1^, B_2g_, and Ag^2^ Raman modes as a function of the applied strain.

**Figure 6:**
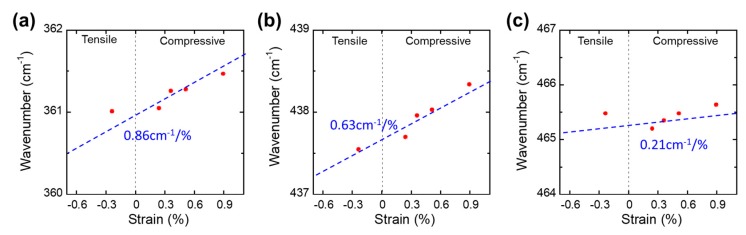
P peaks of (**a**) A_g_^1^, (**b**) B_2g_, and (**c**) A_g_^2^ Raman modes as a function of the applied strain.

After we confirmed the crystalline quality and thickness of BP layers, we performed a strain–Raman relationship spectral study to investigate the Raman shifts under different uniaxial strain conditions. On the basis of the results shown in Figure 2 and Figure 3, the thickness of BP for the strain-Raman relationship spectrum study was found to correspond to 2–3 layers. In order to accurately measure the changes in the Raman spectrum under uniaxial strain conditions, we employed convex and concave molds that have different curve radii ranging from 110 mm to 20 mm, which corresponded to uniaxial strains of up to 0.89% of the tensile strain (for the convex mold) and up to 0.24% of the compressive strain (for the concave mold). The strain-dependent characteristics of the A_g_^1^, B_2g_, and A_g_^2^ modes are shown in Figure 5. While the three different modes showed the same qualitative behavior with respect to the applied strains and exhibited a linear increase (blue shift), the rate of increase was different for each phonon mode. In order to examine the degree of peak shifting, the peak shift as a function of strain was plotted, as shown in Figure 6. The peak shifting values of A_g_^1^, B_2g_, and A_g_^2^ were measured to be 0.86 cm^−1^ /Δε, 0.63 cm^−1^/Δε, and 0.21 cm^−1^/Δε, respectively. The out-of-plane A_g_^1^ mode resulted from the opposing vibrations of the top and bottom P atoms with respect to each other within the same layer. The B_2g_, and A_g_^2^ modes were associated with the in-plane vibration of the P atoms in different directions [34,35]. Therefore, it is reasonable to assume that the A_g_^1^ mode was slightly more sensitive under bending conditions, because the crystal distortion in the vertical direction was more than in the horizontal direction under uniaxial strain. However, these measured slopes were smaller than in the other BP strain studies. It is speculated that the difference derives from the vibrational interaction and light diffraction between BP and the substrate, which was also observed in the other two-dimensional materials [36].

## 4. Conclusions

In summary, we have systematically studied the Raman vibration of BP transferred onto a Ge-coated PDMS substrate, which provided a much higher BP contrast in BP compared to the uncoated substrate and allowed us to investigate much thinner layers of BP directly on a flexible substrate. The Raman spectra taken from several BP layers with different thickness revealed that the clear peak shifting rates for the A_g_^1^, B_2g_, and A_g_^2^ modes were 0.15 cm^−1^/nm, 0.11 cm^−1^/nm, and 0.11 cm^−1^/nm, respectively. Also, the full width at half maximum (FWHM) of all three Raman phonon modes increased as the thickness of the BP layer decreased. Particularly, the peak of the A_g_^2^ mode increased faster than those of the A_g_^1^ and B_2g_ phonon modes, indicating that Raman spectra provide a simple way to identify the number of layers of BP. Using this parameter to identify the 2–3-layered BP, a stain–Raman relationship spectrum study was conducted with a maximum uniaxial strain of 0.89%. The peak shifting values of the A_g_^1^, B_2g_, and A_g_^2^ modes by uniaxial strain were measured to be 0.86 cm^−1^/Δε, 0.63 cm^−1^/Δε, and 0.21 cm^−1^/Δε, respectively. Therefore, the phonon mode peak shifting is a good indicator to gauge strain information.
